# Embracing the comparative approach: how robust phylogenies and broader developmental sampling impacts the understanding of nervous system evolution

**DOI:** 10.1098/rstb.2015.0045

**Published:** 2015-12-19

**Authors:** Andreas Hejnol, Christopher J. Lowe

**Affiliations:** 1 Sars International Centre for Marine Molecular Biology, University of Bergen, Thormøhlensgate 55, Bergen 5008, Norway; 2 Hopkins Marine Station, Department of Biology, Stanford University, 120 Oceanview Blvd., Pacific Grove, CA 93950, USA

**Keywords:** animal phylogeny, homology test, nervous system evolution, brain, longitudinal nerves, phylogenetic systematics

## Abstract

Molecular biology has provided a rich dataset to develop hypotheses of nervous system evolution. The startling patterning similarities between distantly related animals during the development of their central nervous system (CNS) have resulted in the hypothesis that a CNS with a single centralized medullary cord and a partitioned brain is homologous across bilaterians. However, the ability to precisely reconstruct ancestral neural architectures from molecular genetic information requires that these gene networks specifically map with particular neural anatomies. A growing body of literature representing the development of a wider range of metazoan neural architectures demonstrates that patterning gene network complexity is maintained in animals with more modest levels of neural complexity. Furthermore, a robust phylogenetic framework that provides the basis for testing the congruence of these homology hypotheses has been lacking since the advent of the field of ‘evo-devo’. Recent progress in molecular phylogenetics is refining the necessary framework to test previous homology statements that span large evolutionary distances. In this review, we describe recent advances in animal phylogeny and exemplify for two neural characters—the partitioned brain of arthropods and the ventral centralized nerve cords of annelids—a test for congruence using this framework. The sequential sister taxa at the base of Ecdysozoa and Spiralia comprise small, interstitial groups. This topology is not consistent with the hypothesis of homology of tripartitioned brain of arthropods and vertebrates as well as the ventral arthropod and rope-like ladder nervous system of annelids. There can be exquisite conservation of gene regulatory networks between distantly related groups with contrasting levels of nervous system centralization and complexity. Consequently, the utility of molecular characters to reconstruct ancestral neural organization in deep time is limited.

## 1. Morphology, molecules and early nervous system evolution: the early years

The advent of molecular biology and developmental genetic tools has ushered in a new set of comparative data with great potential to impact our understanding of body plan evolution. Long-standing questions about the evolution and origins of animals had been the exclusive realm of comparative morphologists and palaeontologists. Many competing hypotheses had reached an impasse owing to the problems of establishing unambiguous anatomical homologies based on comparative morphology [1]. The amazing diversity of fossils from a variety of deposits with exquisite soft body preservation has led to unprecedented insights into the organization of body plans of animals, close to the origin of the bilaterians [2]. However, molecular clock data are largely in agreement that the origin of the Bilateria predates the Cambrian by a substantial margin [3–5], indicating that there remains significant uncertainty about the evolutionary origins of bilaterians [6,7]. The identification of stem groups remains a contentious issue, and the majority of fossils at the base of the Cambrian are already attributable to crown groups [2,8]. The strange macrofossils from the Ediacaran are believed by some to represent stem bilaterians, whereas others believe them to be representatives of a now extinct lineage of early animals [9]. Early trace fossils are all that remain of small worm-like animals long before the Cambrian, which likely were bilaterians, but the morphology of the burrows leaves plenty of ambiguity to the level of complexity of the animals that left them [10–12].

Molecular biology has provided a rich new set of data to address classical hypotheses of phylogenetic relationships and the deep ancestry of bilaterians. Some of the most compelling recent comparative datasets have come from developmental biology related to ectodermal patterning, and particularly to the patterning of the nervous system [13–15]. The original observations began with the similarities in the expression of *Hox* genes in collinear domains during centralized nervous system development in mouse and fly [16], but went on to reveal further similarities in anterior patterning: genes such as *otx* and *pax6* and a growing list of transcriptional factors and signalling ligands [17–23]. Even 30 years later, the original observation that the CNS patterning of mouse and *Drosophila* share fundamental early patterning similarities is fascinating. Subsequent close examination of a broad range of transcription factors has revealed very close patterning similarities between arthropods and chordates in the patterning of the CNS. This extends not only to structural similarities but is also backed up by functional studies; in mouse knockdowns of *otx*, the resulting mutant animals are entirely lacking a forebrain and midbrain [24,25]. This phenotype was partially rescued by the *Drosophila* homologue *otd*, further building a case for similarities in the molecular construction of a CNS in both flies and mouse [26,27]. As evidence grew from studies of the anterior–posterior (A/P) axis, speculation about the molecular players that define the position of the CNS in both flies and vertebrates began to further build a compelling case of a conserved suite of genes involved in molecular patterning of the nervous system [13,19,20]. Again in flies and vertebrates, similarities in the mechanisms that define the position of the CNS on either the dorsal or ventral side of the body plan, respectively, revealed some fundamental patterning similarities: bone morphogenic protein (BMP)/chordin signalling is involved in defining the region of the ectoderm that will give rise to the CNS [28]. In both cases, broad activation of BMP signalling represses the formation of a CNS and localized expression of BMP antagonists is required to represses the antineuralizing effects of BMP, defining the region along the dorsoventral axis that the CNS will form [29,30]. In flies, the region is on the ventral side, and on the dorsal side in chordates [28,31,32]. This inverted molecular patterning mechanism that defines the formation of the neuroectoderm in both flies and vertebrates resulted in the re-emergence of a classical hypothesis of axis inversion originally proposed by Geoffroy Saint-Hilaire in 1822 [33] and championed by Anton Dohrn [34]. This hypothesis proposed that the dorsoventral axis of vertebrates and arthropods are essentially the same if arthropods are flipped over on their back; all the organ systems line up; ventral heart, dorsal nerve cord and dorsal axial musculature [28,31,35]. Finally, further in the dorsoventral dimension, later patterning similarities in the mediolateral patterning programme of the neural plate and ventral nerve cord were revealed [36,37].

The combined observations of molecular patterning similarities in both the dorsoventral and A/P axes during the formation of the CNS of both arthropods and chordates have led to the prevailing hypothesis that the ventrally centralized nerve cords of arthropods may be homologous with the dorsally centralized medullary cord of vertebrates, and thus present as such in the last common ancestor of Bilateria (in ‘evo-devo’-jargon often referred to as ‘Urbilatarian’) [13,17,28]. Further support for this hypothesis has come from a series of elegant papers from Detlev Arendt's laboratory from the spiralian lineage [37,38]. *Platynereis dumerilii* is an errant polychaete annelid that has become the most established model species to represent spiralians [39]. Particularly striking is the close similarities in the extent of D/V mediolateral patterning of the annelid nervous system with that of vertebrates, and the vertebrate pallium [36,37]. These results suggest that similarities in molecular patterning are strongly connected to the morphological outcome and to the cell type composition of the neural organ systems. The interwoven complexity of the molecular patterning systems with the elaborated neural structures is highly suggestive for a common origin of both and is in favour of a single evolution of a complex nervous system in the stem bilaterian lineage.

A morphologically and molecularly tri-partitioned brain connected to a ventral CNS present in the last common ancestor of protostomes and deuterostomes also implies secondary reduction in animal lineages that have a much simpler organization of their nervous system—for example, anterior basiepidermal nerve rings and lateral neurite bundles that lack perikarya [13,14].

It is extremely challenging to distinguish between hypotheses of homology and homoplasy in structures that are only present in distantly related species such as insects, annelids and vertebrates and are only superficially similar. When we use molecular genetics as a suite of characters to test hypotheses of CNS homology, it is imperative that we have both a broad understanding of the role of molecular genetics in the development of contrasting neural architectures, a detailed analysis of structural correspondence and a robust phylogenic framework to map neural and molecular characters. If loss of nervous system complexity is common in many lineages, then are the highly conserved molecular patterning systems also secondarily simplified to give rise to the less complex neural architectures? Can we detect traces of the hypothesized ancestral complex neural structures in secondarily simplified nervous systems? Alternatively, is it possible that morphologically complex neural architectures such as condensed nerve cords and partitioned brains are fascinating cases of homoplasies driven as response to similar ecological and life-history selective pressures?

The answers to these questions are slowly beginning to emerge as lineages that lack a complex nervous system are being studied using molecular methods, indicating that there is not a one-to-one correspondence between expression similarities and morphological structure. Furthermore, new phylogenomic approaches provide a more robust phylogenetic framework that helps discriminate between homology and homoplasy of structures that have been carefully analysed with advanced morphological and molecular methods.

In this review, we highlight the importance of the comparative approach and recent findings that provide an opportunity for a critical appraisal of previous homology statements. In addition, we describe how recent progress in molecular phylogenies facilitates testing of homology hypotheses and call into question our ability to precisely reconstruct ancestral neural anatomies from the sparse phylogenetic sampling of molecular genetic data.

## 2. Nervous system diversity and its molecular patterning

Any reconstruction of the deep ancestry of morphology requires a comprehensive comparative approach [40,41]. In order to have confidence in our abilities to reconstruct ancestral morphologies in deep time from molecular genetic data, we have to have a clear understanding of (i) the relationship between conserved gene regulatory networks and the wide range of morphologies they regulate; and (ii) a clear understanding of the phylogenetic relationships between the species used to generate molecular genetic data.

Currently, our understanding of the relationship between conserved developmental genetic networks and the evolution of neural architectures is based on a very biased sampling of bilaterian neural architectures, represented by highly centralized and complex CNS organization [13–15]. These animals are characterized by complex life histories and strongly cephalized sensory structures, integrated with complex motor outputs, and only represent a fraction of the extraordinary diversity of bilaterian nervous systems. In order to evaluate fully the utility of molecular approaches for reconstructing ancestral neural architectures, it is essential to investigate the role of these conserved networks during the development of a wider diversity of neural architectures of bilaterians [42]. Bilaterian nervous system organization spans the gamut from a broad basiepithelial plexus with only subtle condensations in the case of xenoturbellids and nemertodermatids [43,44], to vertebrates and arthropods with strongly partitioned CNS and peripheral nervous system (PNS) [45]. However, there are many groups with elements of both a dispersed epithelial plexus and centralized elements in the form of neurite bundles and anterior brains or ganglia [1,14,46]. Are these nervous systems patterned by the same conserved gene regulatory networks, exhibiting the same exquisite relative expression domains along both dorsoventral and A/P axes, or are the simpler neural architectures defined by a degenerate form of the gene regulatory programme?

## 3. A case study: the molecular genetics of A/P axis patterning in hemichordates

**Figure 1.**
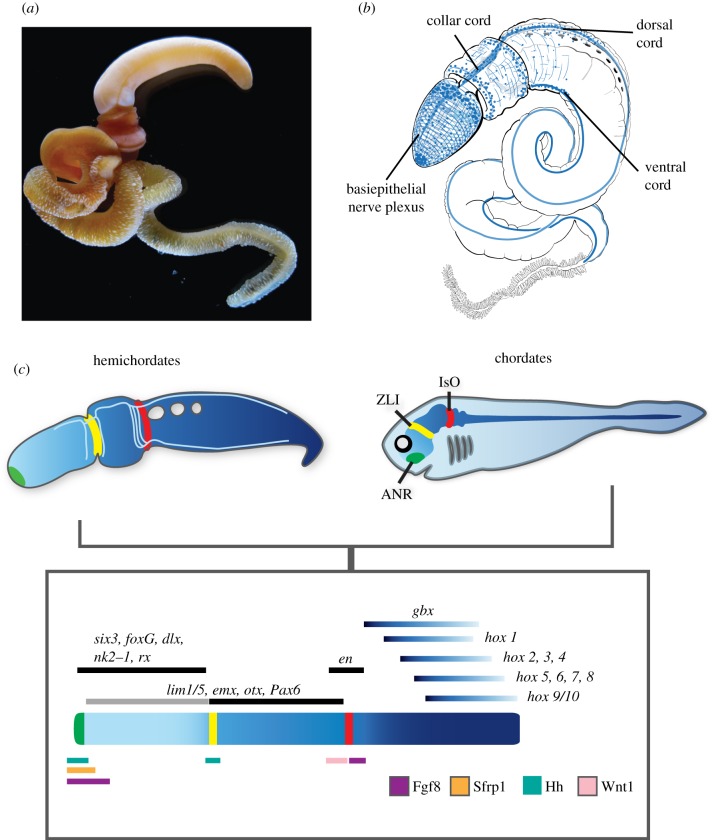
Hemichordate nervous system anatomy and conserved developmental programmes between chordates and enteropneusts. (*a*) An adult enteropneust *Saccoglossus kowalevskii*. (*b*) Schematic of the organization of the nervous system of an adult enteropneust showing a broad epithelial plexus and two nerve cords; one dorsal and one ventral. (*c*) Schematic of the developmental genetic similarities between enteropneusts and vertebrates during early A/P ectodermal patterning. Blue shading represents similarities in the regional expression of orthologous transcription factors between the phyla, and green, yellow and red stripes represent homologous genetic programmes for local signalling centres: ANR (anterior neural ridge); ZLI (zona limitans intrathalmica); and IsO (isthmus organizer), respectively. *en*, *engrailed*. Figure modified from Pani *et al.* [51].

Hemichordates are the sistergroup to echinoderms and this clade, together with the chordates, form the Deuterostomia [47–50]. The two clades of hemichordates are the enteropneusts and pterobranchs [48]. The majority of information on patterning and neural organization comes from the enteropneusts, which are divided into two main lineages, one with direct development and the other with indirect development and a long-lived larval phase (figure 1*a*). The organization of the enteropneust nervous system is characterized by both centralized elements and a pervasive basiepithelial plexus [52–55] (figure 1*b*). The two cords have been a source of speculation about their potential homologies with the dorsal nervous system in chordates [54,56,57]. The dorsal cord extends from the collar down to the anus. In its most anterior extent, in the collar, the cord is internalized by a morphogenetic process that resembles chordate neurulation [53,54,57–59]. The remaining length of the cord is superficial and an extension of the plexus [52]. The dorsal cord connects to a ventral cord via a nerve ring in the posterior collar (figure 1*b*). The ventral cord extends posteriorly from the collar nerve ring down the length of the trunk. The basiepithelial nerve plexus is pervasive in the proboscis and collar, but is most prominent in at the base of the proboscis (figure 1*b*). The plexus extends throughout the animal, but the number of cell bodies drops off significantly in the trunk [52,53].

The organization of the nervous system reflects the life history of the animals, which burrow in sand and mud and feed by both detritus and filter feeding. A brain is absent and there are no centralized sensory organs. The nervous system is a good example of a more modest organization to compare with the highly centralized examples from arthropods and chordates. A series of molecular genetic studies have now been carried out, mainly on the direct-developing species *Saccoglossus kowalevskii* [60]. The most detailed characterization has been in the determination of the patterning of the A/P axis, and largely in relation to the patterning of the vertebrate neuraxis (figure 1*c*) [51,61]. Despite the major differences in the organization of their nervous systems, the relative expression domains of conserved transcription factors are well conserved between vertebrates and hemichordates [62]. Transcription factors that pattern the forebrain of chordates such as retinal homeobox (*rx*), *six3*, *foxG*, *nk2–1* and *dlx* are expressed broadly in the proboscis ectoderm of hemichordates reflecting the organization of the basiepithelial neural plexus (figure 1*c*). Markers of midbrain such as *emx*, *otx*, *Pax6* and *lim1/5* are largely localized in the same circumferential epithelial domain, but further posteriorly into the collar ectoderm, and markers of hindbrain *engrailed* (*en*) and *gbx* are localized in the anterior trunk (figure 1*c*). *Hox* genes are first localized in the anterior trunk down into the posterior embryonic domain, again expressed in broad ectodermal domains in early embryonic stages, and then in later juvenile stages some *Hox* genes become localized to the nerve cords [63,64]. In summary, the relative expression domains down the A/P axis of key regulators of the neuraxis in vertebrates is matched in almost perfect register by the expression of their orthologues in hemichordates, despite the fundamental differences in the organization of the nervous system [61]. The similarities are not limited to many of the basic transcriptional similarities shared broadly with protostomes, they also share the localized ectodermal signalling centres that define the CNS of all vertebrates; the anterior neural ridge (ANR), the Zona limitans Intrathalamica and the isthmus organizer [51,65]. These developmental modules in the CNS are defined by secreted ligands and were thought to be innovations of vertebrates, associated with the assembly of a more complex nervous system. However, their characterization in hemichordates suggests that they form part of the developmental ectodermal scaffold that originated deep in deuterostome evolutionary history—or earlier—and have been modified along each lineage to pattern an array of contrasting morphologies shaped by the specific life histories of the radically different body plans of the deuterostome subtaxa [61]. It is likely that these networks also pattern not only the CNS but the ectoderm more generally, as in hemichordates, echinoderms and chordates, these genes are expressed not only in the nervous system, but throughout the epidermis [13,66,67]. So despite the contrasting neural architectures of deuterostomes, the same suite of genes is involved in patterning in the ectoderm. This suggests that over macroevolutionary time frames, the A/P gene regulatory network has been quite flexible in patterning deuterostome ectodermal derivatives. The disparity of the nervous system architectures in the deuterostome clades Echinodermata, Hemichordata, Chordata (and possibly Xenacoelomorpha [68]), and the lack of a clear understanding of the ancestral nervous system architectures of the outgroups Protostomia and Cnidaria, make it difficult to draw conclusions about the ancestral morphology of the ground pattern of the deuterostome nervous system. Without a comprehensive characterization of the role of these regulatory networks and their connection to the wide diversity of neural organization more broadly in bilaterian groups, our ability to extrapolate ancestral neural architecture from ancestral patterning networks is speculative at best [15,51,62,66].

## 4. Progress in inferring molecular phylogenies provides an emerging phylogenetic framework

Fundamental for comparative biology is a phylogenetic framework on which characters can be mapped and that allows the determination of their distribution and thus the polarity of characters (direction of evolutionary change) [40,41,69]. Furthermore, a reliable phylogeny is fundamental for the congruence test of homology hypotheses that allows us to discriminate between the homology or homoplasy of a character (see below and also [2]).

The homologizations of bilaterian morphological characters over long evolutionary distances, such as segmentation, partitioned brains and nerve cords, have suffered from the absence of a solid phylogenetic framework to rigorously test them. Continued improvements over the past 30 years have resulted in more reliable molecular phylogenies, and the remaining unsolved questions comprise a lower number of alternative hypotheses that can be now specifically tested [6].

The attempts for a systematization of animals have seen dramatic changes in the approach since the historical Aristotle's ‘Historia Animalium’ and Linneus' ‘Systema Naturae’. Before Willi Hennig's revolutionary ‘*Phylogenetic systematics*’ [69,70], taxonomic relationships were dependent on the intuition of the researcher, and were often followed by a subjective series from simple to complex and estimations about the likelihood of the evolution of morphological characters. Furthermore, evaluations of the importance of selected characters such as coeloms (‘Coelomata’), segmentation (‘Articulata’) and development (Protostomia and Deuterostomia) for the reconstruction of the relatedness of animals resulted in different—often incongruent—topologies.

Willi Hennig provided the scientific foundation for reconstructing evolutionary relationships between organisms and thus the basis for all phylogenetic reconstructions since. However, the methods for reconstructing animal relationships themselves have undergone changes [71]. Initially, morphology delivered the only basis for phylogenetic inferences [72]. Advances in methodology allowed more detailed descriptions of morphology, but tree building seemed to have reached an impasse, with often equally likely competing hypotheses. With the advent of molecular sequencing, it became clear that protein and nucleic acid sequences contain evolutionary information [73]. A new source of data became available for phylogenetic inference that is largely independent of morphology. After overcoming the first hurdles [74–76], the ribosomal RNA molecules 18S and 28S changed the view of animal relationships [77,78]. The seminal work by Aguinaldo *et al*. [79] and Halanych *et al.* [80] gave surprising assemblages: nematodes grouped together with arthropods and lophophorate taxa allied with Annelida and Mollusca.

These ‘new’ animal relationships accompanied the rise of ‘Evo-Devo’ as a new discipline [81]. The discovery of similar gene expression in similar organ systems, together with the establishment of the large animal clades Ecdysozoa, Lophotrochozoa and Deuterostomia, delivered promising new data to test long-standing speculations about origins of major organ systems and reconstructions of the last common ancestor of arthropods and vertebrates [82,83]. Molecular biology seemed to provide answers to many debated zoological problems. In order to further test the proposed reconstruction of a complex last common ancestor of arthropods and mouse—the so-called ‘Urbilaterian’—further species were selected to more broadly represent bilaterians: the annelid *P. dumerilii* was chosen not only because it was an established laboratory animal [84] representing Lophotrochozoa, but also because it was proposed to represent the best proxy for the hypothetical ‘Urbilaterian’ [39]. The body plan of this errant polychaete is defined by segmentation, appendage-like structures (parapodia) and a condensed ventral nervous system with a brain. The presence of a biphasic life cycle with a ciliated larva that develops through the spiral cleavage programme was—at least by some researchers—claimed to be ancestral for the protostomes [85]. At that time, the internal phylogeny of the Lophotrochozoa^1^ remained polytomic and thus did not exclude the possibility that annelids are representatives of early branching lophotrochozoans, which could indeed hint that annelids show multiple ancestral characters [77,81]. The claim that annelids share many characters with the bilaterian stem species implies also the secondary simplification of groups such as gastrotrichs, platyhelminthes, gnathiferans [81].

The field of molecular phylogenetics continuously improved its methodology and increased the taxon sampling [86]. Increased taxon sampling of 18S and 28S molecules repositioned obscure taxa such as chaetognaths and acoels to key positions of the metazoan tree. Acoels have been proposed to be the sister group to bilaterians, replacing cnidarians as the most informative bilaterian outgroup [87,88], breaking up the simultaneous appearance of the bilaterian characters nephridia, coeloms, mesoderm, CNS, heart and the one-way gut into an evolutionary sequence, thus challenging some hypothetical scenarios of bilaterian evolution such as the enterocoely hypothesis [89–92]. Additionally, the placement of direct-developing, deuterostomic and unsegmented chaetognaths as sister group of all remaining protostomes questions the proposed homology of segmentation and larvae across Bilateria [93,94].

Further development of computational algorithms and advances in sequencing technologies transformed molecular phylogenetics, from the targeted PCR approach isolating a couple of molecular loci, into phylogenomics using expressed sequence tags and transcriptomes to build matrices that are built upon 1000 different molecular loci [95,96]. This approach led to improved resolution in many parts of the animal tree of life [3]. The chordate ancestor ‘proxy’ Branchiostoma was displaced from sister group status to the vertebrates by urochordates [97] and for the first time, long-standing morphological groupings such as the molluscs, annelids and platyhelminths received molecular support [98].

Despite problems associated with the analysis of large datasets such as systematic errors, paralogy issues and variable informativeness of genes [99], progress has been made in nearly all parts of the tree of life since the first large-scale phylogenomic analyses of animal relationships were published [98]. Further development of the methodology and larger taxon sampling demonstrate the potential to resolve the position of even the most problematic taxa and to address long-branch attraction (LBA) artefacts caused by fast-evolving sequences and rapid radiations [100–102]. This has led to very surprising arrangements in the animal tree of life, such as the sister-group relationship of ctenophores to all remaining animals [98,102–105] and for the first time provides insights into the relationships within the Ecdysozoa and Spiralia (with Lophotrochozoa rendered as subtaxon of Spiralia) [100,106,107].

Although some questions still remain open—e.g. the placement of the Acoela and the internal phylogeny of Lophotrochozoa—an emerging phylogenetic framework is allowing zoologists and evolutionary biologists to map morphological and molecular characters to test long-standing hypotheses of animal evolution that challenge the prevailing views on the early evolutionary origins of bilaterians animals [6]. It is an exciting time to be a comparative biologist, and it is clear that the improved resolution of animal relationships gained from phylogenomics will help resolve many of the current debates of morphologists and evo-devo researchers, and also open up new horizons for future research.

## 5. The phylogenetic framework and testing hypotheses of homology

Increased taxon sampling not only helps to resolve animal relationships, but also leads to a better understanding of the evolution of morphology and its underlying molecular mechanisms. The extension of developmental studies to representatives of taxa at key phylogenetic positions has led to fundamental insights into the role of transcription factors and signalling pathways in the evolution of morphology, as well as insights into genome evolution. However, comparisons of characters over large evolutionary distances, with sparse phylogenetic sampling and the lack of a phylogenetic framework, can only lead to very vague hypotheses about homology [108–111].

**Figure 2.**
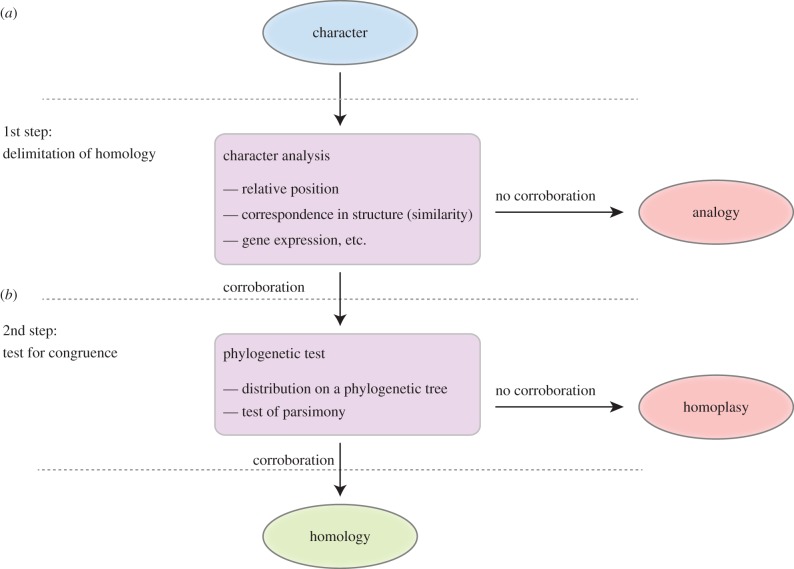
General procedure for testing homology hypotheses. The test of homology is a two-step process in which the homology hypothesis has to pass two definite tests. After the definition of the character, the character is analysed according to criteria such as relative position, correspondence in structure, similarity (but see [112,113]) and ontogeny (*a*) (but see [114–116]). When corroborated, this leads to the formulation of the ‘primary homology’ [117] or ‘potential homology’ [118] hypothesis, otherwise the character is an analogy [113]. In a second step (*b*), the homology hypothesis is tested for congruence using a phylogenetic (‘cladistic’) test for parsimony on the basis of a phylogeny. If the homology hypothesis fails this test, it is a homoplasy. Drawing modified after Rieppel [40] and Richter [119].

Formulation and testing of homology hypotheses of morphological and other characters are an essential part of comparative biology [40] and start with the selection of the character and the collection of indicators for their potential (or ‘primary’) homology (figure 2*a*). This process, also referred to as delimitation of homology, uses different criteria such as the distinct position with respect to other body regions, and correspondence in structure [117,120,121] (‘similarity’, but see [122]). Gene expression and gene regulatory network analysis falls into the ‘similarity’ criterion.

Remane's homology criteria contain, beside position and similarity, also genealogical origin (ontogeny) [123; see also 124]. Ontogeny as a criterion for homology was highlighted as problematic as early as 1894 by Wilson [114] based on the fact that different developmental pathways can still produce homologous structures [115,116].

After the identification of potential homologies, these have to be tested in a second step for congruence within the context of a phylogenetic framework [40,112,119–122,125] (figure 2*b*). It is not sufficient to accumulate data about the similarity, position, and gene expression patterns of a character to claim homology, without a test for congruence that follows in a second step. This step is essential to discriminate between homology and homoplasy of a character. Only after legitimation using a parsimony test based on a phylogenetic tree, can the potential or ‘primary’ homology become a ‘secondary’ homology [117]. De Pinna [117] brings up the example of the bat wing and bird wing—which are common textbook examples for illustrating concepts of homology because they are homologous as vertebrate forelimbs, but homoplasic as ‘wings’—and asks the question; what would we conclude if bats were the sister group to birds? We would probably conclude that wings are also homologous and reconstruct a wing in their last common ancestor, including the underlying gene regulatory network.

It should be clear that the test for congruence is an essential second step—after the careful structural comparison—to avoid premature homology statements. It is largely owing to the uncertainty in metazoan phylogeny that nearly all ‘Evo-Devo’ homology hypotheses that homologize structures across large evolutionary distances based on shared molecular patterning could never be tested for congruence.

The recent inferences of the internal topologies of Ecdysozoa and Spiralia provide a framework on which some homology hypotheses can be tested for congruence. We will focus on two hotly debated features of nervous systems—the tripartite brain and the ventral centralization of longitudinal nerves.

### (a) Increased resolution of the internal relationships of the Ecdysozoa and the case of the ancestry of the ‘tripartite brain’

As discussed previously, comparisons of the molecular patterning of the anterior brains of *Drosophila* and vertebrates led to the discovery that homologous genes are expressed in a very similar fashion along the A/P axis of the fly brain and the brain of the mouse [18–20,22,23]. Intriguingly, the gene expression patterns of *otx*, *emx* and *engrailed* (*en*) correlate—at least to some extent—with the morphological subdivisions of the brain regions of both species [18,23]: the vertebrate brain is subdivided into fore-, mid- and hindbrain and the brain of *Drosophila* is tripartite as well, subdivided into proto-, deuto- and tritocerebrum [126]. Fuelled by the results of the functional equivalence of mouse and fly *otx*, *emx* and *en*, which can at least partly rescue loss of function experiments [27,127,128], the morphological tripartite brain has been assigned to the last common ancestor of protostomes and deuterostomes [18]. However, functional equivalence experiments are not very informative for the reconstruction of ancestral brain morphologies since they address the level of the interaction of genes inside the network. For example, the *emx* orthologue of *Caenorhabditis elegans*, *ceh-2*, is able to partly rescue the *Drosophila* mutant, despite the fact that nematodes themselves do not possess a tripartite brain [129].

In this scenario of deep homology of tripartite brains, divergent brain structures, such as commissural, ring-shaped, mono-, di-partite brains, which are present in most other animal groups [1,45,46], are interpreted as multiple cases of loss or reduction [18]. The scarcity of molecular information about brain development of taxa that lack a tripartite brain makes it currently difficult to test the hypotheses from a molecular perspective (see Martín-Durán *et al*. [130]). In order to investigate the deep bilaterian ancestry of the tripartite brain, we have to test hypotheses of homology at the level of morphology.

Recent morphological studies of the nervous system in representatives of several ecdysozoan groups, in addition to progress in resolving the internal phylogenetic relationships, allow us to test for congruence of the homology of the tripartite brain of insects and vertebrates and also to determine the direction of evolutionary change (polarity) of brain morphology.

**Figure 3.**
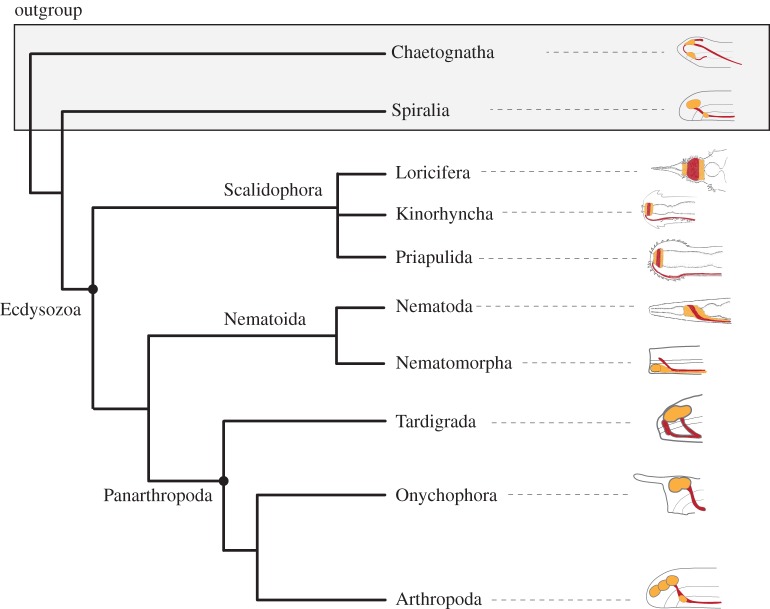
Phylogeny of the Ecdysozoa and schematic of brain structures. The phylogeny is based on a consensus of recent publications that address different nodes in the phylogeny [100,106,134,137]. The brain structures are indicated by line drawings of lateral views of the anterior of representative species (see description and references in §5(a)). The red parts are dominated by neurites and the yellow parts are dominated by the presence of perikarya. Drawings of the lateral views modified from Nielsen [138].

18S and 28S loci did not provide an unambiguous phylogeny for the Ecdysozoa [131]: even with improved taxon sampling, e.g. inclusion of the rare species of Loricifera, trees were prone to LBA artefacts [132,133]. Only with the recent emergence of phylogenomic studies that included broad taxon sampling in Ecdysozoa [100,106,134–136] were more reliable relationships obtained. The emerging topology suggests that the Cycloneuralia (Scalidophora + Nematoida) are paraphyletic, with the Scalidophora forming the sister group to the remaining Ecdysozoa: Nematoida (Nematoda + Nematomorpha) and Arthropoda (Tardigrada, Onychophora, Euarthropoda) [100,106] (figure 3). The two successive branches at the base of the Ecdysozoa comprise small marine groups that are defined by rather simple neuroanatomies.

When mapping brain architectures on this tree, the previous cycloneuralian apomorphy—a ring-shaped neuropil of equal thickness that surrounds the anterior intestine [139,140]—is rendered an apomorphy for all Ecdysozoa (figure 3). But the brain in the cycloneuralian taxa is not as uniform as was first proposed, with divergent architectures in some groups, which mainly differ in the distribution of the somata in the neuropil [140]. It is important in this context to highlight that the somata–neuropil–somata arrangement is a different structure from a tripartite brain of arthropods (and also shows intraphyletic variations) [140]. The scalidophoran groups Priapulida, Loricifera and Kinorhyncha possess the circumoral brain and cycloneuralian arrangement of the neuropile, but in kinorhynchs this ring is interrupted on the ventral side [141]. The complete ring-shaped neuropile in Priapulida can vary in the arrangement of the somata of neurons [140,142], while in the Loricifera so far investigated, the arrangement is more uniform, with an anterior and posterior distribution of the somata [143]. Depending on the internal relationships of the Scalidophora, which currently remains unresolved, either the closed—or ventrally open—ring-like brain is part of the ground pattern of the group.

The two clades of the Nematoida, the Nematoda and Nematomorpha, differ in their brain anatomy (figure 3). While the brain of nematodes forms a compact, ring-shaped neuropil [140,144] and is thus similar to that of the scalidophoran groups, the nematomorphs show only a small anterior condensation so it is unclear if it is an extension of the nerve cord or a separate unit [145,146].

The successive branching of clades that possess a ring-shaped, non-partitioned brain—the Scalidophora and Nematoda—implies that this type of brain was present in the last common ancestor of the Ecdysozoa and provided the starting point for modifications that led to the more complex, partitioned brains of the Panarthropoda (figure 3). If this new topology of the Ecdysozoa is correct, then the presence of the non-partitioned, ring-shaped, circumoral brain in the ecdysozoan ground plan rejects the hypothesis of the homology of the morphologically tripartite brain of arthropods and chordates [18,20,147]. Additionally, in the Panarthropoda, the arthropod outgroups—Tardigrada and Onychophora—render the structure of the tripartite brain of vertebrates and arthropods as homoplastic (figure 3). Recent anatomical studies of tardigrade nervous systems reach contrasting conclusions about the segmental organization of their brains [148]. One study claims that the brain of a tardigrade is indeed tripartite and proposes homology of these elements with the proto-, deuto- and tritocerebrum of arthropods [149]. Most studies however, cannot detect individual brain clusters or any innervated cephalic appendages [150–154]. Furthermore, developmental studies fail to detect a partitioned anlage of the tardigrade brain and show that the brain develops from a single, ectodermal source that forms a single lobate structure [155,156]. Previous studies of the onychophoran cephalic nerves also come to contrasting conclusions, ranging from a circumoral brain, similar to that of the ‘Cycloneuralia’ [157], or a tripartition proposed to be homologous to the proto-, deuto- and tritocerebrum of the arthropods [158]. Taking the innervations of the cephalic appendages into account, Mayer *et al.* [159] seems to have demonstrated the bipartition of the onychophoran brain of which the anterior part is possibly homologous to the protocerebrum and the posterior part to the deutocerebrum. A recent follow-up study using retrograde fills of pharyngeal nerves of the onychophoran seems to confirm this bipartition and proposes the presence of a compound brain that evolved by convergent fusion of the ganglia [160]. The possibility of a tripartite origin of the bipartition of the onychophoran brain is excluded from the currently debated scenarios since there is no evidence from morphology [148,160]. The situation in the Chelicerata is ambiguous, since the pygnogonids—the possible sister group to the remaining chelicerates—seem to have a similar, bipartite structure [161]. This is contrasting the presence of a tritocerebrum in the Euchelicerata, e.g. in Xiphosura [162].

In summary, based on the current understanding of the internal topology of the relationships of the ecdysozoan clades, the morphological subdivision of the arthropod brain into proto-, deuto- and tritocerebrum is likely a derived condition for the arthropod members of the Ecdysozoa. However, even the reconstruction of a tripartite brain for the arthropod stem species is unclear and currently a matter of debate.

We propose that the subdivision of the arthropod brain into proto-, deuto- and tritocerebrum is likely an evolutionary novelty in the arthropod lineage and the structural similarities to the partition of the vertebrate brain can be viewed as homoplasies. The current topology of the ecdysozoan relationships makes it problematic to argue for multiple cases of loss of a tripartite brain, since one would have to assume that the stem lineage would have retained such a brain over millions of years without leaving traces in either extant species or the fossil record. It has to be pointed out here that the argumentation for ‘loss’ in comparative biology should be plausible by the character distribution on phylogeny as well. For example, only the phylogenetic topology allows us to state that urochordates have undergone the loss of some morphological characters [68,97], or that several lineages of formerly ‘archiannelids’ are reduced [100,163,164].

If the tripartite brain in vertebrates and arthropods represents a case of homoplasy, how do we interpret the similarities on the molecular level? The first step towards an answer is to investigate the role of the conserved genes in species that show divergent structures. It is fundamental for the understanding of the origin of the tripartite arthropod brain to understand the ‘cycloneuralian’, circumoral brain and its underlying molecular patterning. The only scalidophoran representatives that are currently accessible using molecular methods are the species *Priapulus caudatus* and *Halicryptus spinulosus* [165]*.* An investigation of the molecular basis of circumoral brain patterning will be necessary to test whether priapulids share the same patterning mechanisms of arthropods despite the overt morphological differences in brain anatomies (see also [130]).

In case a conserved network is present, a careful study will be necessary to understand what these patterning systems are actually regulating and how this relates to the morphological outcome. This approach will provide insights into the ancestral role of patterning genes during animal evolution and how changes in these networks are expressed in morphological structure.

### (b) Breaking long branches: the paraphyly of ‘Platyzoa’ and its impact on bilaterian nerve cord evolution

The discussions about the origin of a CNS with a ventrally or dorsally condensed longitudinal nerve cord are largely based on the assumption that the ventral rope-ladder-like nervous system present in some annelids (e.g. *P. dumerilii*) and arthropods are homologous [13,14,32,37,166]. This organization is strongly associated with a segmented body plan, since its subdivisions correlate with the individual body segments. However, not all Ecdysozoa and Spiralia possess such a segmented body plan and ventral CNS, and the distribution of these characters in the phylogeny are important to infer the ancestral state.

**Figure 4.**
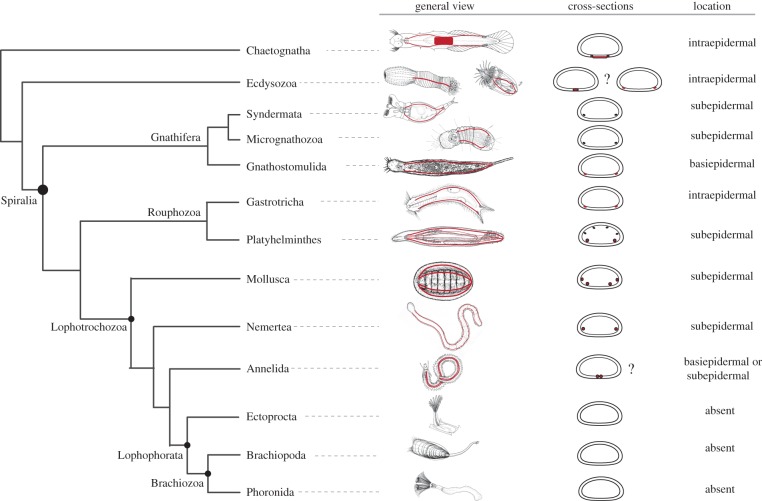
Phylogeny of the Spiralia and the schematic of the location of longitudinal nerves. The phylogeny is based on a consensus of recent publications that address the internal phylogeny of the Spiralia [100,107]. The animal line drawings in the ‘general view’ column illustrate the localization of the main longitudinal nerves for each taxon in red. The schematic mid-body cross-sections in the ‘cross-sections’ column show the location of the main longitudinal nerves in red. The last column ‘location’ indicates the position of the longitudinal nerves in relation to the epidermis.

With the placement of the lophophorate taxa into the protostomes, a long-standing question about the affiliation of brachiopods, phoronids, bryozoans and entoprocts had been solved [80]. Halanych *et al*. [80] delivered the node-based definition of Lophotrochozoa for the last common ancestor of molluscs, annelids and lophophorates and all its descendants. Subsequent studies that include a larger taxon sampling confirmed the lophophorate position inside a taxon that comprises Lophotrochozoa, Gastrotricha, Platyhelminthes and Gnathifera, which together are named Spiralia [86,167]. However, the internal relationships of this assemblage remained unclear, and multiloci and phylogenomic approaches repeatedly recovered the taxon Platyzoa composed out of Rotifera, Gnathostomulida, Gastrotricha and Platyhelminthes as sister to the Lophotrochozoa (reviewed in [3,6,167,168]). The Platyzoa are conspicuous because their long branches indicate rapidly evolving molecular sequences, which suggest that this grouping might be an artefact that is based on LBA [100,107]. Recently, phylogenomic methods have been improved to better address LBA artefacts by using site heterogeneous models [99,169]. New studies that increased taxon sampling in the Spiralia and applied appropriate phylogenomic methods were able to reduce the LBA effect and rendered the Platyzoa paraphyletic into two clades at the base of the Spiralia: Gnathifera and the so-called Rouphozoa (Gastrotricha + Platyhelminthes) [100,107] (figure 4). If this result is robust and is supported by future studies, it has a tremendous impact on our understanding of animal body plan evolution and the evolution of development [170].

The broad-scale topology places two separate groups at the base of the Spiralia that are rather small, simple, interstitial animals—the Gnathifera and the Rouphozoa (figure 4). The relevance of this for recent discussions about animal body plan evolution cannot be underestimated since they all lack segmentation, coeloms, a ventral CNS and larval dispersal stages.

These characters are only found in a subset of the Trochozoa and thus most likely evolved independently during the diversification of the clade. If true, then proposed homologies of many of the complex traits of certain trochozoans to arthropods and chordates are rendered as homoplasies [6]. Below we exemplify and discuss the impact of these new relationships on the proposed homology of the ventrally centralized nervous systems of annelids and arthropods.

The Gnathifera is the sister group to the remaining Spiralia [100,107] and is composed of the Gnathostomulida, Syndermata and Micrognathozoa [171]. The gnathostomulids possess three pairs of basiepidermal, longitudinal nerves (neurite bundles) of which the ventrolateral ones lack perikarya [172–174]. Such paired ventrolateral connectives constitute the major organizational features of the nervous system of micrognathozoans [175,176] and Rotifera [177–179] (figure 4). These lateral nerves are rarely connected by commissures, and in the few cases where they are present, commissures are not to a ganglion-like structure (figure 2).

Gnathifera forms the sister group to Trochozoa (=Lophotrochozoa) and Rouphozoa (Gastrotricha + Platyhelminthes) [100,107]. The gastrotrich nervous system in the trunk is very similar to the Gnathifera and is composed of a single pair of lateral neurite bundles lined by neuronal somata [180,181] The platyhelminth nervous system is usually referred to as a typical orthogon composed of pairs of dorsal, lateral and ventrolateral cords [182]. Recent phylogenomic approaches to solve the internal phylogeny of Platyhelminthes agree upon the split of Cantenulida and Rhabditophora, with the Macrostomida as sister to all remaining Rhabitophora [183,184]. The ‘microturbellarian’ catenulid and macrostomid nervous systems are relatively similar and the comparison with other members of the group allows the reconstruction of the ground plan for the Platyhelminthes [185–187]. This ground pattern comprises a pair of main neurite bundles, which are located laterally in the slightly orthogonal nervous system [188]. These main neurite bundles are partly lined by perikarya and thus can be described as a medullary cord, while some of the dorsal and lateral neurite bundles lack neural cell bodies and are usually referred to as ‘minor cords’ [187,189].

The similarity of the pair of lateral neurite bundles in Gnathifera, Platyhelminthes and Gastrotrichs is striking and suggests that at least such lateral—and not ventrally centralized—longitudinal nerves form the ancestral condition for the Spiralia (figure 4). The remaining clades of the Lophotrochozoa (or Trochozoa) show a variable pattern of the trunk nervous system, and the internal relationships are still not fully resolved. Figure 4 shows the spiralian interrelationships based on recent phylogenetic analyses [100,107], which we use as a preliminary framework to map trunk nervous system architectures to detect the direction of evolution (see above).

In recent years, internal phylogenetic relationships of major trochozoan groups have been addressed using phylogenetic tools (Mollusca: [190,191]; Annelida: [192,193]; Nemertea: [194]). Well-resolved internal relationships allow us to reconstruct ground patterns for different organ systems and can highlight species that are likely to be informative for addressing specific evolutionary questions [195]. Although the internal phylogeny of Lophtrochozoa is not fully settled yet [100], we can make some reasonable approximations to what trunk nervous system architecture was likely present in the stem species of Mollusca, Annelida, Lophophorata and Nemertea: the nervous systems of the lophophorate taxa Brachiopoda, Phoronida, Bryozoa and Entoprocta are characterized by the presence of the lophophore and their sessile lifestyle [46]. The main nervous system is present as a basiepidermal nerve plexus with stronger innervation of the lophophore [1]. Molluscs vary in their nervous system based on their lifestyle, but the ground pattern is likely represented by two pairs of longitudinal nerves—the pedal nerve that innervates the foot and the lateral nerve [196]. These lateral cords are often connected with commissures which led to their description as ‘tetraneural orthogon’ [197,198]. Nemertean trunk nervous systems possess two lateral cords that are internalized—the body also possesses an intraepidermal nervous system [199–202]. The only group in the Spiralia for which a ventrally centralized, rope-ladder-like nervous system composed of two or more cords with ventral ganglia that are connected with ventral commissures has been proposed are the annelids [203,204]. However, the morphological variation of such architecture inside the Annelida is surprisingly variable, and it depends on the internal phylogenetic relationships if the textbook example of such rope-ladder-like ventral nerve cord is even ancestral for the Annelida [203,205]. For example, in the first two separate lineages that are sister groups to all the remaining annelids, the Owenidae + Magelonidae and Chaetopterida [100,163,164,193], the nervous system is still basiepidermal [206] and has been internalized below the musculature during the evolution of other annelid groups possibly multiple times independently.

Altogether, progress in resolving Spiralian relationships allows a reassessment of character evolution in the morphologically diverse Spiralia. This analysis does not support the hypothesis that annelids exemplify the ‘Urbilatarian’ [207] representing ancestral character states for the Spiralia. The most recent research findings suggest that annelids are a highly specialized group that evolved a complex ventral nerve cord likely by elaborating and centralizing lateral cords that are present in most other Spiralian groups. Even the textbook rope-ladder-like ventral CNS that has been studied in great molecular detail in *P. dumerilii* may not be part of the ground pattern of Annelida [203,205]. This is plausible when one considers that annelids are the only spiralian taxon that changed locomotion by cilia to undulating movements of a segmented body and use of appendages (parapodia). Likewise, the evolution of the prominent segmented body plan of annelids was connected to this change [208] as well as the elaboration of the ventral musculature [209].

Similar to the proposition that the tripartite brain as a neural structure is homologous to the vertebrate tripartite brain, the ventrally centralized nerve cord in annelids and arthropods—when tested for congruence—is likely a homoplasy. These observations also impact the hypothesis about the homology of the vertebrate dorsal nervous system and the ventral nervous system of arthropods (see above). Since the Protostomia are the outgroup of Deuterostomia, the reconstruction of the stem species of the Protostomia impacts what we infer as ancestral for the Deuterostomia.

## 6. Conclusion and outlook

If the tripartite brain and the highly centralized trunk nervous system of vertebrates and arthropods are examples for fascinating homoplasies, how do we interpret the similarities on the molecular level? This fundamental question about previous attempts to homologize structures across large evolutionary distances based on similar patterning genes relates not only to neural characters, but also to other organ systems, and their tissues and cell types. If the underlying genetic programme is identical at the level of signalling cascades and transcription factors, what is it that shapes the morphological differences? A recent study that compared the expression patterns of the priapulid digestive tract with that of *Caenorhabditis elegans* and *Drosophila melanogaster* shows that although the digestive tracts of all three animals develop using vastly different modes and differ also in their final morphology, the underlying network of transcription factors shows a high correspondence [210]. This work, and the work on ectodermal patterning and evolution in *Saccoglossus* [61,62], show that the reconstruction of ancestral morphologies based primarily on molecular genetic data is rife with difficulties [211]: similar—and likely conserved—gene regulatory networks seem to be able to regulate a very divergent morphological outcome over macroevolutionary timeframes, so care has to be taken when using these networks as evidence for morphological homology, especially if the conclusions drawn on ancestral character states based on developmental genetic datasets are in conflict with conclusions drawn from morphological studies [212].

In order to reconstruct the early evolution of animal groups and to understand changes in evolution, we need to invest resources into reconstructing character states at different nodes in the animal tree of life. We also need a better appreciation of both the phylogenetic position and basic biology of so-called ‘minor’ groups, since they have the potential to help us understand the direction of evolutionary change in deep time. Recent progress in resolving animal relationships also demonstrates that attempts to homologize superficially similar structures without knowledge about the intermediate taxa can lead to premature conclusions about organ system evolution. Improved knowledge about animal relationships, and the continued expansion of developmental and morphological studies into representatives of formerly neglected groups, will lead to a better understanding of how genetic information regulates morphological structures, and how this changes over macroevolutionary timeframes to give rise to the astonishing animal diversity we observe in nature.

## Endnote

1 We refer here to Lophotrochozoa in its original definition by Halanych *et al*. [80]. In the following, we use the taxon name Spiralia, since recent phylogenies render Lophotrochozoa as subclade of Spiralia.
